# Supplementary material for: Inhibition of the Heat Shock Protein A (HSPA) Family Potentiates the Anticancer Effects of Manumycin A
Source: Cells. 2021 Jun 7;10(6):1418. doi: 10.3390/cells10061418 (PMC8229576; doi:10.3390/cells10061418)
Supplement: Supplementary file 1 [file cells-10-01418-s001.zip › cells-1238600-supplementary.pdf]

Table S1. List of antibodies used in Western blot analyses

	Host / Clonality	Clone	Catalog Numer / RRID	Source	Dilution WB
**FT-α**	Mo/M	D-5	sc-374262 / AB_10989066	Santa Cruz Biotechnology, Inc., Dallas, USA	1:1000
**FT-β**	Mo/M	B-7	sc-46664 / AB_669044	Santa Cruz Biotechnology, Inc., Dallas, USA	1:1000
**panRas**	Mo/M	C-4	sc-166691 / AB_2154229	Santa Cruz Biotechnology, Inc., Dallas, USA	1:1000
**Sp1**	Mo/M	E-3	sc-17824 / AB_628272	Santa Cruz Biotechnology, Inc., Dallas, USA	1:1000
**HSPA1**	Mo/M	C92F3A-5	ADI-SPA-810-F / AB_311860	Enzo, Life Sciences, Famingdale, NY, USA	1:5000
**HSPA2**	Ra/M	EPR4596	Ab108416 / AB_10862351	Abcam, Cambridge, UK	1:5000
**HSPA5**	Mo/M	A-10	Sc-376768 / AB_2819145	Santa Cruz Biotechnology, Inc., Dallas, USA	1:1000
**HSPA6**	Mo/M	165f	ADI-SPA-754 / AB_10615942	Enzo, Life Sciences, Famingdale, NY	1:3000
**HSPA8**	Mo/mAb	B-6	sc-7298 / AB_627761	Santa Cruz Biotechnology Inc., Dallas, TX, USA	1:7500
**HSPA9**	Mo/M	D-9	Sc-133137 / AB_2120468	Santa Cruz Biotechnology, Inc., Dallas, USA	1:2000
**HSF1**	Ra/P	-	ADI-SPA-901 / AB_10616511	Enzo, Life Sciences, Famingdale, NY, USA	1:2000
**Phospho HSF1 (S326)**	Ra/M	EP1713Y	EP1713Y / AB_1267208	Abcam, Cambridge, UK	1:3000
**β-actin (HRP)**	Mo/M	AC15	A3854 / AB_262011	Merck KGaA, Darmstadt, Germany	1:20000
**Secondary**					
**Anti-Mo IgG (HRP)**	Go/P	-	AP124P / AB_90456	Millipore, Billerica, MA, USA	1:5000
**Anti-Ra IgG (HRP)**	Go/P	-	AP132P / AB_90264	Millipore, Billerica, MA, USA	1:2000

Abbreviations: RRID, Research Resource Identifier; M, monoclonal; P, polyclonal; Mo, mouse; nd, no data; Ra, Rabbit; Go, Goat; HRP, horseradish peroxidase

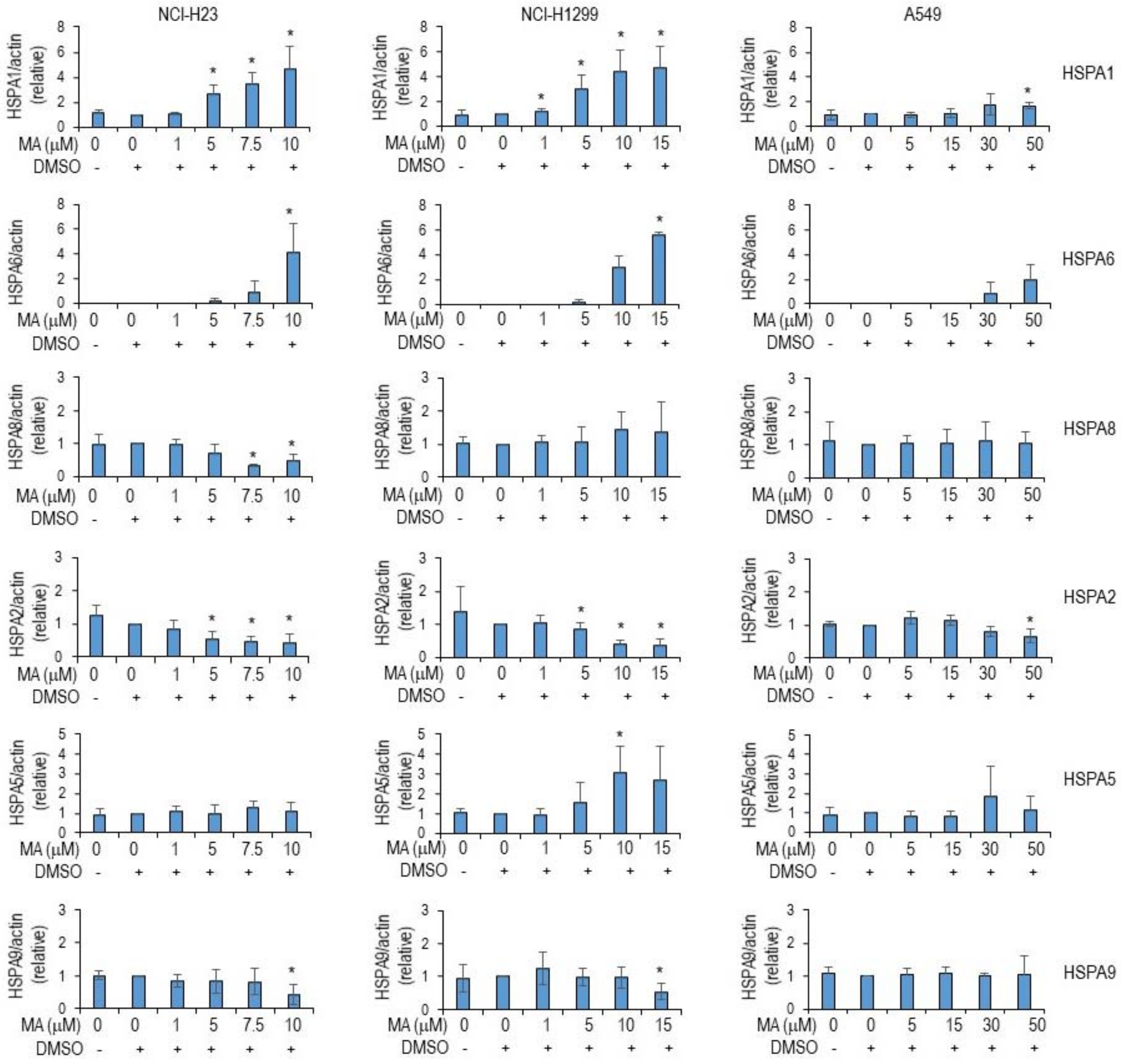

Figure S1. Effects of manumycin (MA) on the protein levels of HSPA paralogs. Densitometric analysis of immunoblots that were showed in Figure 2a was performed using ImageJ Software. Each graph shows results (mean ± SD) generated from at least three independent immunoblots. The relative protein level is shown after normalization to reporter protein level (actin), in case of HSPA6 as HSPA6/actin ratio. Statistical significance was calculated in relation to cells exposed to DMSO (Dimethyl Sulfoxide) solvent.

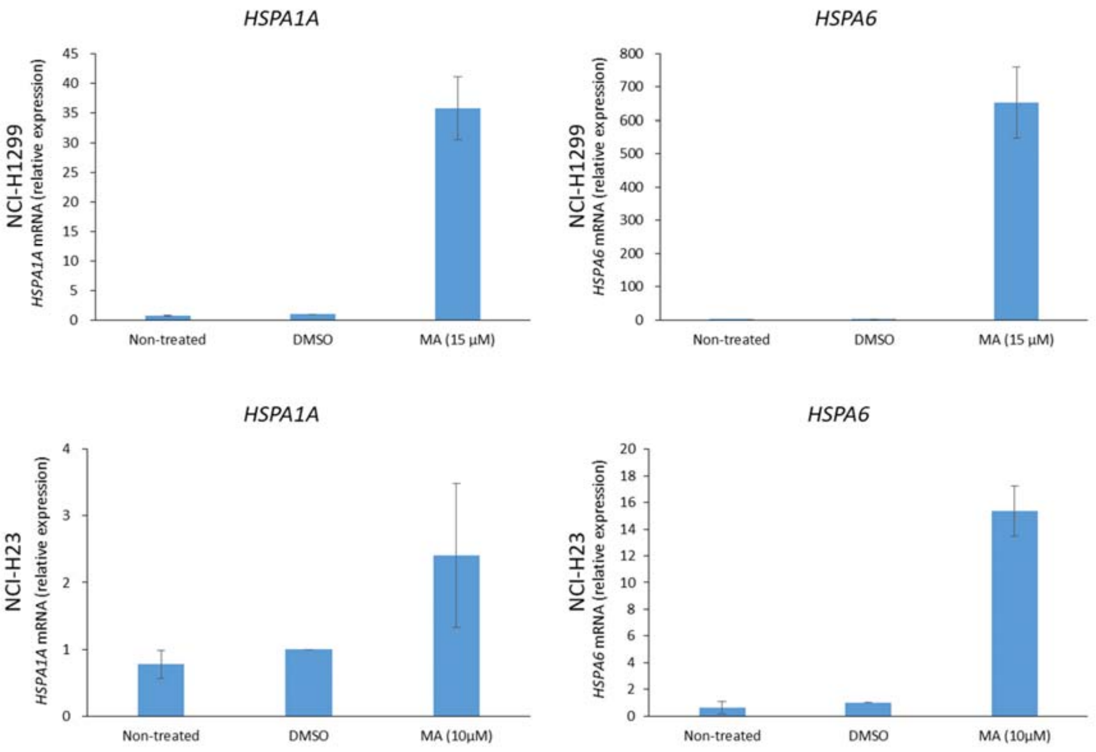

Figure S2. Effects of MA treatment on the mRNA expression levels of *HSPA1A* and *HSPA6* genes in lung cancer cells assessed by RT-qPCR. Cells were exposed to MA for 24 h, cells were harvested and total RNA was isolated using Nucleospin RNA Plus kit (Macherey-Nagel, Germany) according to manufacturer's protocol. cDNA synthesis and RT-qPCR reactions were performed according to our standard protocols [20]. Exemplary result (out of two independent repeats) showing relative increase in mRNA expression is shown. Relative expression was calculated using the 2^($^{-\Delta\Delta Ct}$) method and normalized to the reference index, obtained by calculating the geometric mean of *RPL13A* and *B2M* reference gene expression. Sequences of gene-specific starters are as follows: HSPA1A_F, 5′ AGCTGGAGCAGGTGTGTAACCC 3′; HSPA1A_R, 5′ AAAAACAGCAATCTTGGAAAGGCCC 3′; HSPA6_F, 5′ TCCTGCCCTTCAGAGATGAACT 3′; HSPA6_R, 5′ AAGAGGATGAACCGCCCTCC 3′; RPL13A_F, 5′ CCCTACGACAAGAAAAAGCGG 3′; RPL13A_R, 5′ TCCGGTAGTGGATCTTGGCT 3′; B2M_F, 5' CTGGGTTTCATCCATCCGACA 3'; B2M_R, 5' GTCTCGATCCCACTTAACTATCTTGG 3'.

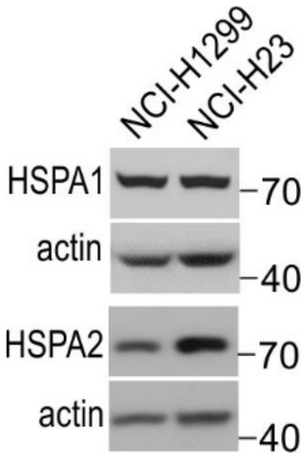

Figure S3. The basal levels of HSPA1 and HSPA2 protein in NSCLC cell lines. Total protein extracts were blotted and detected using respective primary antibody (as indicated in Table S1). Representative immunoblots are shown (n ≥ 3) and actin was used as a protein loading control. The numbers on the right side of immunoblots indicate molecular weight of the protein size marker.

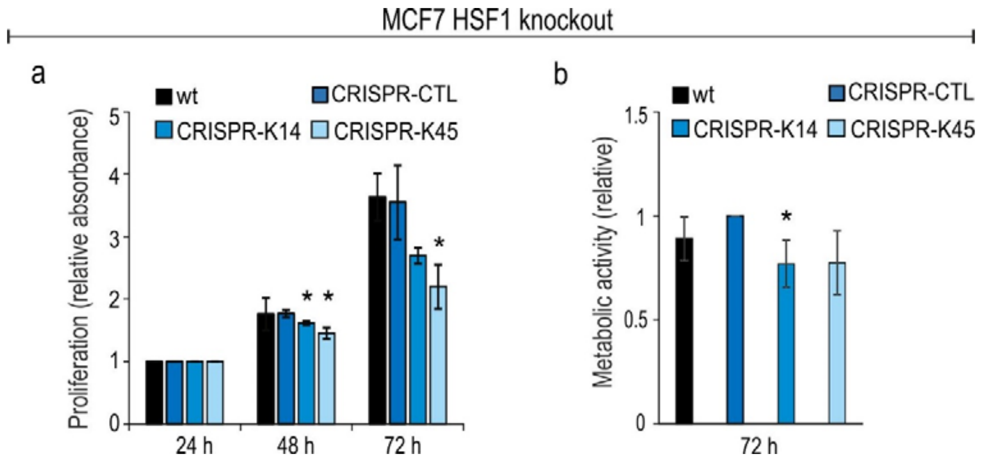

Figure S4. Effects of HSF1 knockout on the proliferation of MCF7 cells. (a) Cell proliferation at 24, 48, 72 hours (h) was determined by the crystal violet staining assay. Cells (2 × 10^4 cells per well) were seeded and cultured in 12-well plates. At the indicated time cells were washed with PBS, fixed in cold methanol, and rinsed with distilled water. Cells were stained with 0.1% crystal violet for 30 min, rinsed with distilled water extensively, and dried. Cell-associated dye was extracted with 1 ml of 10% acetic acid. Aliquots (200 µl) were transferred to a 96-well plate and the absorbance was measured at 595 nm (Synergy2, BioTek). Values were normalized to the optical density at the 24 h time point; all experiments were performed in triplicate at least. **(b)** Metabolic activity was assessed by MTS assay after 72 h of continuous cell growth under standard culture conditions. Results (mean ± SD, n = 3, each in three technical repeats) are expressed relatively to control (CRISPR-CTL).
